# Workplace Health Promotion and COVID-19 Support Measures in Outpatient Care Services in Germany: A Quantitative Study

**DOI:** 10.3390/ijerph182212119

**Published:** 2021-11-18

**Authors:** Felix Alexander Neumann, Elisabeth Rohwer, Natascha Mojtahedzadeh, Nataliya Makarova, Albert Nienhaus, Volker Harth, Matthias Augustin, Stefanie Mache, Birgit-Christiane Zyriax

**Affiliations:** 1 Midwifery Science—Health Services Research and Prevention, Institute for Health Services Research in Dermatology and Nursing (IVDP), University Medical Center Hamburg-Eppendorf (UKE), Martinistr. 52, 20246 Hamburg, Germany; e.rohwer@uke.de (E.R.); n.makarova@uke.de (N.M.); b.zyriax@uke.de (B.-C.Z.); 2 Institute for Occupational and Maritime Medicine (ZfAM), University Medical Center Hamburg-Eppendorf (UKE), Seewartenstr. 10, 20459 Hamburg, Germany; n.mojtahedzadeh@uke.de (N.M.); harth@uke.de (V.H.); s.mache@uke.de (S.M.); 3 Department of Occupational Medicine, Hazardous Substances and Public Health, Institution for Statutory Accident Insurance and Prevention in the Health and Welfare Services (BGW), Pappelallee 33/35/37, 22089 Hamburg, Germany; a.nienhaus@uke.de; 4 Competence Center for Epidemiology and Health Services Research for Healthcare Professionals (CVcare), Institute for Health Services Research in Dermatology and Nursing (IVDP), University Medical Center Hamburg-Eppendorf (UKE), Martinistr. 52, 20246 Hamburg, Germany; 5 Competence Center for Health Services Research in Dermatology (CVderm), Institute for Health Services Research in Dermatology and Nursing (IVDP), University Medical Center Hamburg-Eppendorf (UKE), Martinistr. 52, 20246 Hamburg, Germany; m.augustin@uke.de

**Keywords:** workplace health promotion, outpatient care, ambulatory care, health and safety, occupational health, caregivers, COVID-19, support measures, break room, digitisation

## Abstract

Working conditions in the care sector, especially under the increased strain during COVID-19, make it difficult for outpatient caregivers to adhere to health-promoting behaviours. Research on workplace health promotion (WHP) and COVID-19 support measures in outpatient care services is limited. The aim of this pilot study was to characterise the current situation of WHP and COVID-19 support measures in outpatient care services and to explore how to offer WHP support measures targeted for a specific group. A web-based cross-sectional survey was conducted with outpatient caregivers (*N* = 171) in northern Germany. The results showed that 60.2% of the study participants were offered WHP support measures, with significantly higher work engagement when WHP support measures were available (*Z* = 4279.50, *p* < 0.01) and that 77.2% received specific support from their employer during the COVID-19 pandemic. Although spending a break in a break room was significantly associated with longer breaks as compared with being in a car (*Z* = 39.10, *p_adj._* = 0.01), a break room was neither available in all outpatient care services, nor did it appear to be feasible. Overall, WHP in outpatient care services is insufficiently covered. In order to be able to offer WHP support measures that are targeted to outpatient caregivers, cooperation among local care services, feasibility, and digital measures should be examined.

## 1. Introduction

Due to demographic changes, the proportion of Germans aged 65 and older will rise to 5.6 million by 2060 [1]. This will lead to an increase in the number of people in need of care and, thus, also in the number of nursing staff required [2]. There is already a shortage of skilled workers in the care sector today [2]. Additionally, the high occupational health burden due to shift work, time, and performance pressure [3], and an increased potential for social conflict when dealing with patients [4], has made it difficult to adhere to health-promoting behaviours [5]. Consequently, this can result in health impairments [6], as reflected in the German statistics on incapacity to work [7]. With an average of 22.9 days of sick leave in 2018, nurses missed eight more working days per year than the national average for all professionals, mainly due to musculoskeletal and mental illnesses [7]. Although it is known that occupational requirements can increase the risk of physical and mental illnesses [8], few studies have analysed the burden of diseases among outpatient caregivers as well as their resources [9]. However, studies performed during the COVID-19 pandemic have found that nursing staffs faced new job demands in their work activities [10,11]. These include policy measures, such as hygiene regulations, and masking requirements [10], as well as new daily tasks, such as taking patients’ temperatures [11]. Therefore, negative stress reactions such as depressive symptoms have been reported [10]. Workplace health promotion to strengthen resources might be more necessary than ever, since the nutrition, physical activity, stress, and sleep of outpatient caregivers have also been negatively affected by the changes due to the pandemic [12]. However, during the pandemic, the focus of healthcare services was more on adapting to the COVID-19 pandemic and less on workplace health promotion [13]. Nevertheless, by offering workplace health promotion support measures, healthcare services may be able to improve their employees’ health, as well as increase the attractiveness of businesses for employees, which is important in view of the shortage of skilled workers [14]. Furthermore, studies among inpatient nurses have shown that high health-promoting behaviours were associated with higher job satisfaction and lower work-related stress [15,16], and general health was associated with higher job satisfaction and higher work engagement [17].

To make workplace health promotion measures available, a special feature and challenge in the working conditions of outpatient caregivers is that patient care takes place at the patients’ homes. Route planning is complex, especially in large cities with intense traffic [18]. Delays due to the traffic create conflicts with patients’ expectations that outpatient caregivers are on time [19]. A lack of time has already been described in care research [20,21] and was identified as a decisive factor for the non-use of intervention services [20]. Insufficient time resulting in skipping or shortening breaks and taking lunch in a car have been reported by outpatient caregivers as coping mechanisms that save time [22]. Additionally, spending breaks in a car encourages unhealthy “snacking” which has also been described as a strategy to cope with stress [23].

Although detrimental health behaviour among care workers is known [24,25,26,27] and has been attributed to high physical and mental strain [23,28], research about workplace health promotion in outpatient care services is lacking studies on interventions targeted to this specific group. One reason for this could be the overall complex framework conditions [29,30]. Practical starting points for workplace health promotion could be the availability of a break room in the healthcare service [31] or a digital implementation, for example, health apps [19]. However, it is not known how many outpatient caregivers have a break room available and whether they use health apps at all.

While the relevance of special health promotion for outpatient caregivers is undisputed, no special workplace health promotion measures in outpatient care services have been developed in Germany thus far [32]. When it comes to existing measures developed for inpatient caregivers in Germany, the programmes have mostly focused on stress management, but neglected important aspects of preventive health behaviours, such as exercise and nutrition [30]. An exception to this is the project QualiPEP, which aimed at strengthening health literacy as well as the development of quality-assured behavioural and structural preventive health promotion measures in the areas of nutrition, exercise, cognitive resources, psychosocial health, and violence prevention [33]. In outpatient care services, a comprehensive and sustainable project for workplace health promotion was successfully implemented in Austria [34]. This could be a guide for successful implementation of workplace health promotion in Germany, but it needs to be adapted to the work situation and national healthcare system [21]. Furthermore, model projects such as Astra plus and PA-TRES have aimed to address stress management, smoking cessation, and a healthy lifestyle among nurses in training [35,36]; however, in order to achieve long-term success, sustainable establishment of health promotion in the everyday professional life of outpatient caregivers is necessary.

Therefore, the aim of this pilot study was to characterise the current situation of workplace health promotion in outpatient care services and to explore how to apply WHP support measures targeted for outpatient caregivers.

## 2. Methods

### 2.1. Design, Participants and Recruitment

We aimed to include 199 outpatient caregivers from northern Germany in our online questionnaire, based on a sample size calculation performed with G*Power (*α* = 0.05, *β* = 0.20, *d* = 0.20) [37]. For the recruitment of participants, 367 outpatient care services were contacted by telephone and via the e-mail distribution list of the Hamburg regional group of the Federal Association of Private Providers of Social Services. A total of 253 outpatient care services agreed to hand out the flyer about the study to their employees, while 114 outpatient care services declined participation. The reasons for refusal varied, but mainly lack of time, lack of interest, and reluctance to participate in the survey were mentioned. A cumulative number of 607 prospective participants accessed the online questionnaire between May 2020 and February 2021, of which *n* = 171 (28.2%) initiated and successfully completed the questionnaire, *n* = 315 (51.9%) dropped out, and *n* = 121 (19.9%) did not enrol. Within our recruitment period, COVID-19 developed through various pandemic phases, protection measures and laws constantly evolved, while outpatient caregivers were exposed to ongoing occupational stress, which lowered the participation rate. We estimated that as more different phases of the COVID-19 pandemic were included in our recruitment period, the higher the potential bias could become. In February 2021, when another change in the pandemic phase seemed to be approaching, the data collection was stopped in order to keep potential bias as low as possible, although the calculated sample size was not completely reached.

The online questionnaire, which was pretested by other researchers and one outpatient caregiver, informed about the purpose, anonymity, and voluntary nature of the study, as well as obtained informed consent. Eligibility criteria for participation were that respondents were outpatient caregivers in northern Germany with at least six months of work experience and at least 25 h of work per week.

### 2.2. Variables, Measures and Processes

We addressed our research to the following issues: (a) measures for workplace health promotion and their usage in outpatient care services, (b) work engagement of outpatient caregivers who were offered workplace health promotion measures as compared with colleagues who were not offered any workplace health promotion measures, (c) the role of a break room and its link to health promotion in outpatient care services, (d) the role of apps’ usage for obtaining health information or health tracking, and (e) support measurements in times of COVID-19. Participants’ characteristics gathered by the questionnaire were gender, age, cultural origin, highest education completed, and information about the work situation, i.e., whether the respondents were supervisors and worked in shifts (Supplement Table S1). According to the focus group interviews with experts from the Hamburg care sector, which were conducted in January 2020, the following workplace health promotion measures were identified as known for outpatient caregivers: nutritional counselling, cooking courses, breakfast with colleagues, free fruits, cost reductions and vouchers for fitness studios, sports classes (e.g., fitness or yoga at workplace), back school courses, provision of company bicycles, courses on the topic of maintaining health, stress management courses, and courses on dealing with violence/self-defence and addiction prevention (e.g., cessation seminars for smokers) [19]. Self-developed questions were used to ask about the offer, desire, use, and benefit of the respective measures. Furthermore, based on the information from this question, participants were subdivided into a dichotomous grouping variable named “work health promotion measures offered (yes/no)”. To assess work engagement, we used three items “I am full of energy in my work”, “I am enthusiastic about my work” and “I am completely absorbed in my work” from the validated German version of the Copenhagen Psychosocial Questionnaire [38], which were answered on a 5-point Likert scale ranging from never (1) to always (5). Based on the responses, we obtained sum scores ranging between 3 and 15 points. In order to characterise break behaviour, questions were asked about the availability of a break room, the average length of breaks over the last four weeks, the place(s) where breaks are usually spent, as well as whether and how COVID-19 had an influence in this context. Furthermore, questions on the use of health apps and the provision of support measures in dealing with the COVID-19 pandemic were self-developed. The complete list of self-developed questions used is shown in the Supplement Table S2.

### 2.3. Statistical Analysis

Participants’ characteristics are shown as percentages, means, and standard deviations. Shapiro–Wilk tests indicated that several variables were not normally distributed; therefore, parametric test procedures were used for the statistical analysis. Spearman’s ρ was applied for correlations. Comparisons of two groups were performed by using the Mann–Whitney U test. Three or more groups were compared with the Kruskal–Wallis test. The significance level was set to *α* = 0.05. SPSS Statistics (version 26.0; IBM Corp., Armonk, NY, USA) was used for statistical analysis.

## 3. Results

### 3.1. Participants

The study sample consisted of 171 outpatient caregivers. Male (33.3%) and female (65.3%) caregivers were not equally represented. Overall, 132 participants (77.2%) had German parents and 39 (22.8%) participants had at least one parent who was not born in Germany. More detailed information on study participants is provided in Table 1.

### 3.2. Workplace Health Promotion

**Figure 1:**
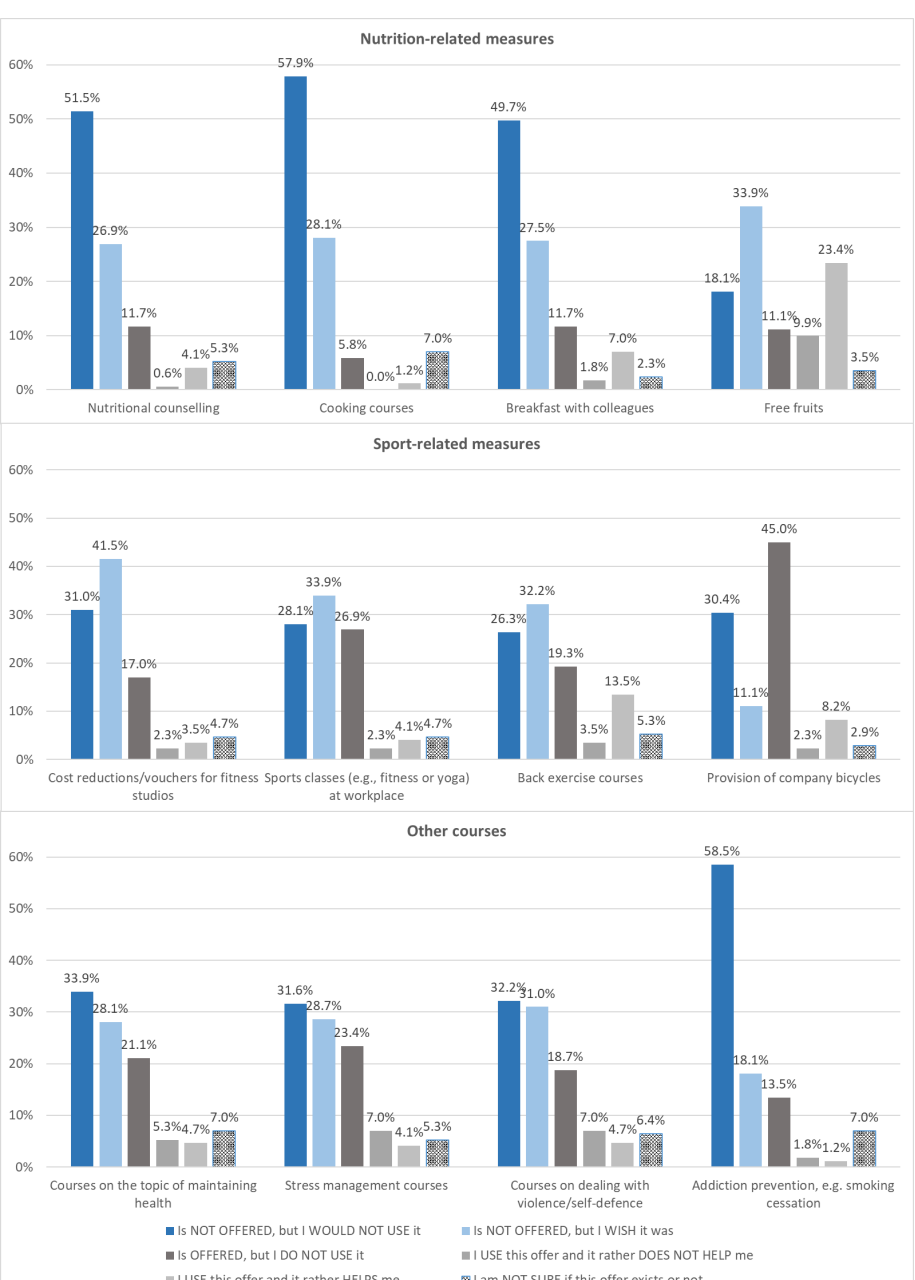
Evaluation of offer, desire, use, and benefit of twelve health promotion measures.

While 103 participants (60.2%) stated that they were offered workplace health promotion measures prior to the COVID-19 pandemic, 68 (39.8%) participants were not offered any of the questioned health promotion measures at all. Figure 1 shows the offer, desire, use, and benefit of twelve questioned health promotion measures. Most frequently offered were company bicycles (55.5%), free fruits (44.4%), back exercise courses (36.3%), and other courses such as stress management (34.5%), sports (33.3%), and maintaining health (31.1%). However, only a small proportion of the participants used most measures. In the free text field, in which the participants were asked to name desired workplace health promotion measures, nine outpatient caregivers stated they would like to have better professional support. Specifically, they stated further training on working time management, supervisions, and internships in other areas of care. Furthermore, an active break offer (four participants), better route planning (one participant), an ergonomic design of the workplace (one participant), wellness vouchers and company outings (one participant) were mentioned. As reasons for not participating in the health promotion activities offered, outpatient caregivers stated, in the free text field, that they did not have the time due to private commitments (eight participants), that these activities competed with privately conducted health promotion activities (e.g., fitness classes) (six participants), or that outpatient caregivers did not feel the need to attend information sessions because they considered themselves to be well enough informed (four participants). However, there were also criticisms of the existing measures because they “don’t change anything anyway” (three participants), are not in line with needs (three participants), mostly take place during duty hours (two participants), are too far from the workplace (one participant), and are too expensive (one participant).

### 3.3. Work Engagement among Outpatient Caregivers

**Figure 2:**
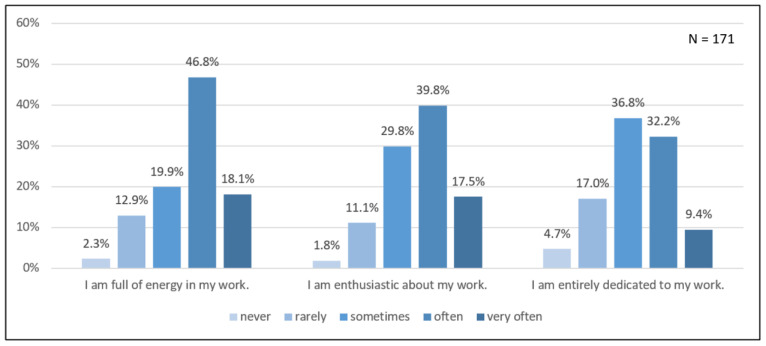
Aspects of work engagement among outpatient caregivers. The responses (*N* = 171) of the three items were correlated (*r* = 0.60, *p* < 0.01), and Cronbach’s alpha (*α* = 0.82) indicated a good internal consistency.

About two-thirds of the participants indicated that they were often or very often full of energy at work (Figure 2). The results for enthusiasm for work were similar, as the majority reported being enthusiastic often or very often. The results for work dedication were lower, with more than half of the participants sometimes, rarely, or never being dedicated at work.

Furthermore, a group comparison showed that the total score of work engagement was significantly higher for outpatient caregivers who stated that health promotion measures were available at their workplace (*Z* = 4279.50, *p* < 0.01) as compared with their colleagues who responded that they did not to have access to any measures.

### 3.4. Potential Approaches for Workplace Health Promotion Measures

Approximately two-thirds of the participants stated that they had a break room available at their workplace, while less than one-third did not have a break room available. Regarding break locations, 21.6% of the participants reported a break room, 16.4% of the participants reported outside such as in the park, 13.5% of the participants reported a car, and 15.8% of the participants reported other places such as a café or supermarket (Supplement Figure S1). Another 32.7% of the participants reported several of the aforementioned locations for breaks, and they thus spend their break at different break locations. The average self-reported actual break length in the past four weeks was M (SD) = 23.9 (12.23) min; thus, presumably, for a relevant sample proportion, this was below the legally prescribed break duration.

The longest break duration was reported by outpatient caregivers who stated they usually spent their break in a break room. Participants who reported spending their break in a break room took significantly longer breaks than those who spent their breaks in a car (*Z* = 39.10, *p_adj._* = 0.01) (Table 2).

Furthermore, the outpatient caregivers were asked whether the changes due to the COVID-19 pandemic had an influence on their break behaviour. Overall, 33.9% of participants answered “yes”, 57.9% of participants answered “no”, and 8.2% of participants responded, “don’t know”. In the free text field, the participants stated that, since the pandemic, breaks were increasingly spent outside or in a car instead of at a client’s place or in a break room with colleagues (eight participants), breaks had become more irregular and spontaneous (six participants), breaks had become less frequent or are omitted due to the high workload (four participants), and breaks were needed more frequently (one participant).

**Figure 3:**
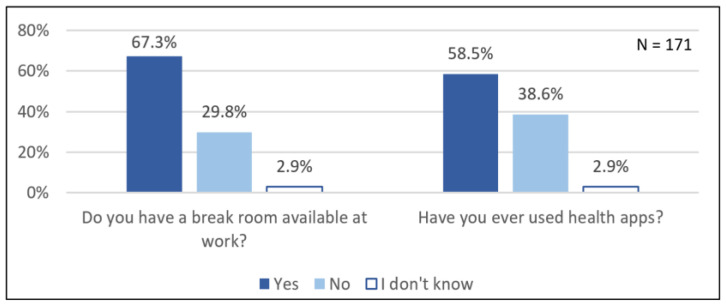
Potential approaches for workplace health promotion.

Regarding the use of health apps, more than half of the outpatient caregivers reported having used health apps (Figure 3). Among the participants who had used health apps, most had used sports or pedometer apps (83% of participants), followed by nutrition (58% of participants), relaxation and stress reduction (43% of participants), and sleep (41% of participants) (Supplement Figure S2).

### 3.5. Support Measures for Dealing with COVID-19

About four out of five participants stated that they received support from their employer in dealing with the COVID-19 pandemic. “No” answers were more frequent at the beginning of the pandemic and decreased over time (Supplement Figure S3). In the free text field, the outpatient caregivers indicated support in the form of protective equipment, such as masks, disinfectants, disposable gloves, and rapid tests (20 participants); advice and information on awareness, such as newsletters and training courses (19 participants); discussions with line managers or other contacts from the company, as well as an anxiety phone queries (six participants); and close involvement of the employees, such as consideration of service requests, appreciation, and co-decision making, as well as good teamwork (three participants).

## 4. Discussion

This is the first study to quantitatively assess the availability and use of workplace health promotion and COVID-19 support measures for German outpatient caregivers during the pandemic, which are both of high importance for caregivers’ health due to the special work settings of outpatient care services and the necessity for direct contact with patients [18]. Our results indicated that COVID-19 support measures and workplace health promotion measures are not routinely available to outpatient caregivers. In addition, different workplace health promotion measures were offered and were insufficiently used. Nevertheless, we found higher work engagement among outpatient caregivers who had access to workplace health promotion measures than those who did not. Furthermore, the average break time was less than 30 min, which suggests that some participants did not make full use of their break time. In addition, not every outpatient care service provided a break room and the participants who replied that they spent their breaks in a break room took significantly longer breaks than those participants who responded that they spent their breaks in a car.

### 4.1. Offers and Use of Workplace Health Promotion Measures in Outpatient Care Services

The challenges of implementing health promotion measures in outpatient caregivers have been sufficiently described [12,18,19,20,21,22,23]. A high workload resulting from a high volume of care and a simultaneous shortage of skilled workers often makes health promotion at the company level secondary [19,21]. Moreover, the provision of workplace health promotion measures seems to be particularly difficult for small and medium-sized enterprises due to financial and organisational aspects [39]; and because of the mobile setting, outpatient caregivers are only present in the care service for a short time, which limits their accessibility to health promotion measures [18]. This is also reflected in the results on workplace health promotion measures for our study sample. The workplace health promotion measures that were included on the questionnaire were all offered, but the quantity and choice of available measures strongly differed among care services. In total, less than two-thirds of all respondents stated that they had access to workplace health promotion measures and the high response rates for “is not offered, but I wish it was” indicate a desire for health promotion measures. Nevertheless, the proportion of outpatient caregivers who responded to participate in workplace health promotion measures was low. For inpatient care, a lack of time during the working hours has already been reported as the most frequent barrier to prevent the use of health-promoting measures [20,40]. However, the diversity of the occupational group in terms of age and cultural background, which is also reflected in our sample, additionally complicates the provision of desired and needs-based measures [30]. Reasons given by our participants included time and performance pressure; the measures do not seem very attractive, successful, or adequate for the needs of outpatient caregivers; as well as local and financial aspects (e.g., too far away from the workplace or too expensive). Furthermore, the use of workplace health promotion measures might have also been impacted due to the additional burden of work during the COVID-19 pandemic, as described by Mojtahedzadeh et al. [10]. Outpatient caregivers were more exhausted by their daily work routine, which consequently might have caused the omission of health-promoting behaviours [41]. Along with that, the participants’ most frequent suggestions for workplace health promotion measures were directed towards optimising work processes, presumably in order to reduce stress. Nevertheless, currently, available workplace health promotion support measures should also be questioned by the employer with regard to relevance, attractiveness, and availability for employees in outpatient care services. Government economic incentives could strengthen the resources of small and medium-sized healthcare enterprises and thus encourage the establishment of workplace health promotion measures [42]. In addition, the responses of outpatient caregivers indicated they desired more professional support such that they could take responsibility for solving their problems themselves. Therefore, the development of new workplace health promotion measures by means of a participatory approach would be promising in order to offer WHP support measures targeted for a specific group in the future [3]. Additionally, it should be taken into consideration that, in order to be able to achieve long-term success, the combination of behavioural and structural preventive measures is recommended [43].

### 4.2. Work Engagement and Workplace Health Promotion

Although outpatient caregivers responded that they had seldom used the available workplace health promotion measures and that these did not meet their needs, we found higher work engagement among outpatient caregivers who had accessed measures at work as compared with those who had not accessed measures. In addition, for inpatient caregivers, it was shown that work engagement was positively related to job satisfaction, work performance, and nurse-assessed quality of care; thus, both outpatient caregivers and those in need of care might benefit by promoting caregivers’ health [44,45]. Furthermore, a study of inpatient caregivers from Turkey found an inverse relationship between job satisfaction and staff retention [46], which, in turn, suggests that the turnover rate of skilled workers from the profession might also be reduced by an increase in work engagement.

### 4.3. Availability and Importance of Break Rooms

About one-third of the participants stated that they did not have access to a break room and the average break time was less than 30 min, which suggests that some participants do not make full use of their break time. In addition, the duration of breaks was longest for those participants who spent their respite in the break room and significantly longer than for those who said they spent their break in a car. Along with this, consciously skipping breaks and taking meals in a car have often been described by outpatient caregivers as coping behaviours for saving time [19,22]. This goes along with a study by the German Federal Institute for Occupational Safety and Health, which found that legally mandated breaks could not be taken due to the amount of work involved [47] and, as a result, developed a checklist for examining the organisation of breaks in care activities [48]. Having a break room close to patients and the arrangement of the break room were judged important in a study on inpatient care [49]; however, these criteria are not always feasible for small outpatient care services. By using a cross-sectional survey among 597 outpatient and inpatient caregivers from 80 geriatric care teams, Wendsche et al. [50] found that the turnover rate of skilled workers was lower when caregivers regularly took breaks with their colleagues. Therefore, it should be examined whether break rooms could be available for a larger proportion of outpatient caregivers, for example, through cooperation among nearby care services.

### 4.4. Use of Health Apps: Is Digitalisation an Opportunity for Workplace Health Promotion?

More than half of the participants in our survey stated they had used health apps in their free time at least once, which showed that some of the outpatient caregivers had an interest in receiving digital information on health-related topics or in self-tracking. Moreover, web-based programmes for muscle relaxation and sports exercises were highlighted as likely to be used among outpatient caregivers [41]. However, it was also reported that the idea of apps would be somewhat rejected by older outpatient caregivers [41]. Nevertheless, looking to the future, web-based workplace health promotion seems to be a promising approach. Knowing that there are digitalisation projects with the aim of optimising outpatient caregiver work processes [51], the possibility should be examined whether and to what extent workplace health promotion can find its way into these platforms. Especially in organising the work processes with networked route planning and service recording, digitalisation is described as a possible solution to some problems, such as skipping breaks due to a lack of time to complete work tasks [51]. Studies on inpatient care services have demonstrated that digitalisation represents an opportunity for the implementation of workplace health promotion, since programmes for stress prevention as well as for occupational health management with integrated optimisation of work processes have been proven successful in randomised controlled trials [52,53,54]. In addition, the COVID-19 pandemic poses a particular challenge for the implementation of workplace health promotion measures, since, for example, infection protection measures such as distance regulations make it difficult to implement group-based offers such as sports courses. In general, considering the COVID-19 pandemic consequences, digital solutions to advance prevention and health promotion should be considered as priorities in workplaces [55].

### 4.5. Support Measures in Outpatient Care in Relation to COVID-19

Surprisingly, only about three-quarters of the participants indicated they had received COVID-19 support measures by their employer. A closer look at the data revealed that especially those outpatient caregivers who filled out the questionnaire at the beginning of the survey, which was a few weeks after the arrival of the pandemic in Germany in March 2020 [56], did not receive any support. This shows that the caregivers and organisations were not prepared for the pandemic situation and, thus, could not offer support to outpatient caregivers; conversely, the shortage of protective equipment and hygiene articles that could be provided became apparent [10]. Protective equipment, such as masks, disinfectants, disposable gloves, and rapid tests, as well as advice and information on awareness, such as newsletters and training courses were named most frequently as support offers. Protective measures or sources of information for adequate protection were particularly relevant in times of COVID-19 [57]. These prevented additional infection among caregiving colleagues. Moreover, caregivers are inevitably in physical contact with their mostly elderly patients, which exposes them to a higher risk as recipients and caregivers can quickly become carriers of the disease to a vulnerable group [58]. Furthermore, a study of German semi-residential and outpatient care facilities reported higher absenteeism among caregivers due to sickness or quarantine, such that 40 min on average of extra work per shift had to be compensated for by healthy colleagues as compared with before the pandemic [59], which resulted from the increased psychosocial burden that healthcare professionals were exposed to [60]. Accordingly, advice and information on awareness, such as newsletters and training courses, are equally important support measures, as Rohwer et al. [61] demonstrated a link between perceived information sufficiency and pandemic-related worries and perceived stress. This also explains why the study participants in our survey mentioned responses such as “discussions with line managers” or “good teamwork” as support measures, although these are not measures in the true sense of the word. 

### 4.6. Strengths and Limitations

Our study was designed to generate exploratory insights for an inadequately studied field of research [30]. Based on a self-conducted expert workshop [19], our online questionnaire was developed to quantify insights generated from this workshop, which are important for the development and implementation of workplace health promotion measures. The use of an online survey tool ensured the completeness of the dataset. In addition, the validity of the data was increased by using pre-adjusted conditional response options in the questionnaire editor, as well as cognitive testing afterwards by two of the authors. Another strength of our survey comes from the diversity of the demographics of our study population, which was achieved by including participants from urban districts of varying social background in a large city in northern Germany.

However, there are also limitations that should be taken into consideration. Due to the COVID-19 pandemic in Germany and the contact restrictions imposed by the German government during the pandemic [62], it was not possible to present our survey or information material to the caregivers in person. Thus, recruitment success was largely dependent on the willingness of care managers to cooperate and on the self-motivation of outpatient caregivers to participate. However, self-motivation to participate in our survey may have been negatively influenced as high professional demands of outpatient caregivers such as shift work as well as time and performance pressure [3] were additionally reported to be increased due to the pandemic [10]. As the launch of our survey coincided with the first COVID-19 pandemic wave in Germany, this led to a low participation rate and high dropout rate. A long study period of ten months was necessary to achieve a satisfactory number of participants. Thus, the recruitment period encompasses different phases of the pandemic, which may have partially influenced response behaviour. For example, responses to the question “Do you receive support from your employer in dealing with the COVID-19 pandemic?” were negative more frequently at the beginning of the survey period than at later points in the pandemic, indicating that care services had not been prepared for this particular situation, but adjustments were made as the pandemic progressed. We had reasonable doubt whether the data quality of the smaller than desired sample was sufficient for modelling; hence, we decided to focus on the meaningful descriptive results. Furthermore, our sample of 171 outpatient caregivers only equals 0.04% of the total outpatient caregiver’s workforce in Germany of *N* = 421,550 [63], whereby the extent of representativeness remains limited. 

### 4.7. Implications for Further Research and Practice

Since there has been little research on workplace health promotion in outpatient care in Germany [30], there are several promising starting points for further research. First, the possibility to use a break room as a linchpin for workplace health promotion should be explored. Equipping a break room with a massage chair has been shown to significantly contribute to reducing stress, blood pressure and heart rate among caregivers in inpatient care [64]. However, it is unknown whether and to what extent the provision of health promotion support measures in a break room would be used among outpatient caregivers. Additionally, a promising measure to apply in outpatient care services’ break rooms would be the principles of nudging [65] but depending on the arrangement and equipment of the break room, it would have to be checked individually how the healthy options could be highlighted. Moreover, in the context of the pandemic, the importance and opportunity for establishing digital services in outpatient care becomes apparent, but it still needs to be investigated how the full potential can be exploited and how digital workplace health promotion can be usefully arranged for outpatient care. In the future, feasibility studies should explore which formats are most promising. In addition, web-based platforms may be a suitable medium to strengthen the health literacy of outpatient caregivers, which could encourage health-promoting behaviours, as well as improve their health, work motivation, and work productivity [29,66]. 

## 5. Conclusions

This is the first quantitative study assessing the availability and use of workplace health promotion and COVID-19 support measures among German outpatient caregivers. In summary, we found a gap in the supply of workplace health promotion measures. However, work engagement was significantly higher among outpatient caregivers who stated that health promotion measures were available at their workplace. Due to work-related time constraints and the diversity of the employee group, a wide distribution and combination of local and digital support offerings for workplace health promotion would be desirable. As a basis for on-site workplace health promotion, a break room has great potential, as it leads to longer breaks, but it was neither available in all outpatient care services, nor appeared feasible for small businesses. Cooperation among local care services is recommended in order to guarantee a health-promoting break culture, which would support the establishment and expansion of support measures for workplace health promotion. Although digitalisation has provided a new starting point that could facilitate successful development and implementation of WHP support measures targeted for a specific group, outpatient care services are, thus far, insufficiently covered and researched for developing evidence-based programmes. 

In addition, our findings show that about four out of five participants received support measures from their employers in dealing with the COVID-19 pandemic, yet some outpatient care services might have taken a long time to adapt. Employers and policymakers should be better prepared for critical situations such as a pandemic in the future, as the provision of protective material and promotion of workplace health constitute important components of occupational safety and health.

## Figures and Tables

**Table 1:** Demographic characteristics.

		*n* (%)
**Gender**		
	Male	57 (33.3)
	Female	112 (65.5)
	Diverse	2 (1.2)
**Age (Years)**		
	18–29	18 (10.5)
	30–39	55 (32.2)
	40–49	40 (23.4)
	50–59	44 (25.7)
	≥60	14 (8.2)
**Origin**		
	Both parents born in Germany	132 (77.2)
	One parent at least born abroad	39 (22.8)
**Highest Education**		
	General secondary school	18 (10.5)
	Intermediate secondary school	87 (50.9)
	Specialised grammar school	21 (12.3)
	Grammar school	45 (26.3)
**Work Situation**		
	Supervisor	62 (35.4)
	Shift work	89 (52.0)

**Table 2:** Length of breaks spent at the different break locations as compared with in a break room.

Break Locations Compared to the Break Room	*n*	M	SD	*Z* ^a^	*p*	*p_adj._* ^b^
Break room	37	27.03	11.93	-	-	-
Multiple responses	56	26.43	12.31	2.82	0.77	1.00
Outdoors (e.g., park)	27	24.39	8.21	10.66	0.36	1.00
Other (e.g., café, supermarket)	28	20.00	12.63	25.87	**0.03**	0.26
Car	23	16.74	12.93	39.10	**<0.01**	**0.01**

Note: *N* = 171; ^a^, Kruskal–Wallis Tests; ^b^, *p* values adjusted by the Bonferroni correction for multiple tests; results directed according to the hypothesis; *p* < 0.05 in bold.

## Data Availability

The data analysed during the current study are not publicly available due to German national data protection regulation. They are available on individual request from the corresponding author.

## Supplementary Materials

The following are available online at https://www.mdpi.com/article/10.3390/ijerph182212119/s1, Figure S1: Break location of outpatient caregivers, Figure S2: Use of health apps subdivided according to health topics, Figure S3: Responses to the question “Do you receive support from your employer in dealing with the COVID-19 pandemic?” over time, *N* = 171, Mann–Whitney U = 1839.5, *p* = 0.007, Table S1: Variables of the study instrument used to survey participant characteristics, Table S2: Self-administered variables of the study instrument used in the analyses.
