# Supplementary material for: Workplace Health Promotion and COVID-19 Support Measures in Outpatient Care Services in Germany: A Quantitative Study
Source: Int J Environ Res Public Health. 2021 Nov 18;18(22):12119. doi: 10.3390/ijerph182212119 (PMC8620567; doi:10.3390/ijerph182212119)

Supplementary Figure Legends

Figure S1. Break location of outpatient caregivers.

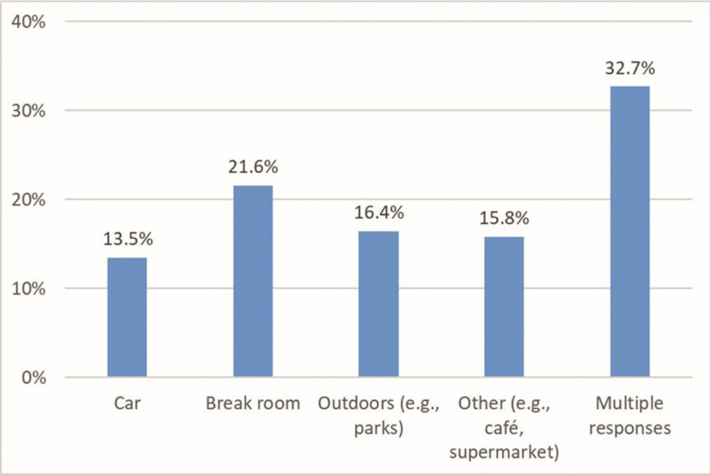

Figure S2. Use of health apps subdivided according to health topics.

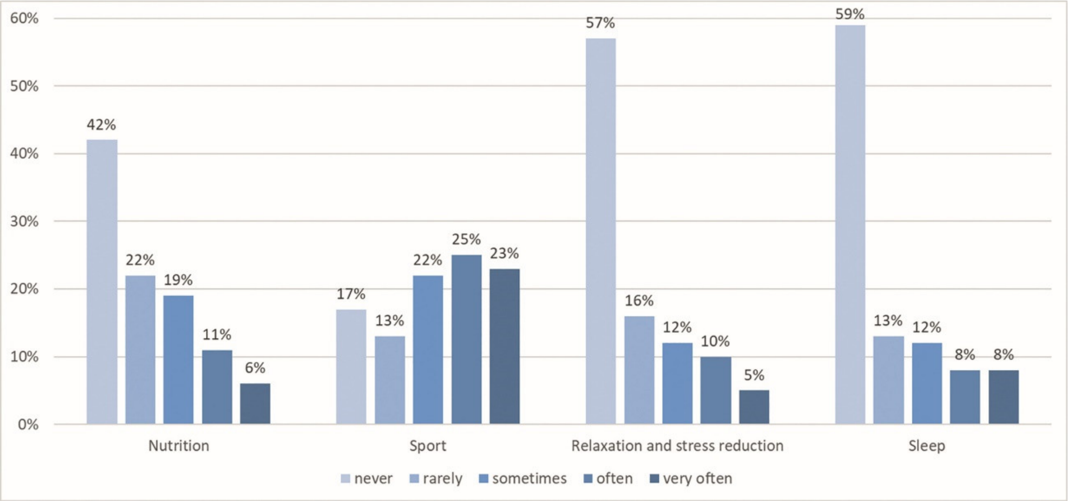

Figure S3. Responses to the question “Do you receive support from your employer in dealing with the COVID-19 pandemic?” over time, N = 171, Mann–Whitney U = 1839.5, p = .007.

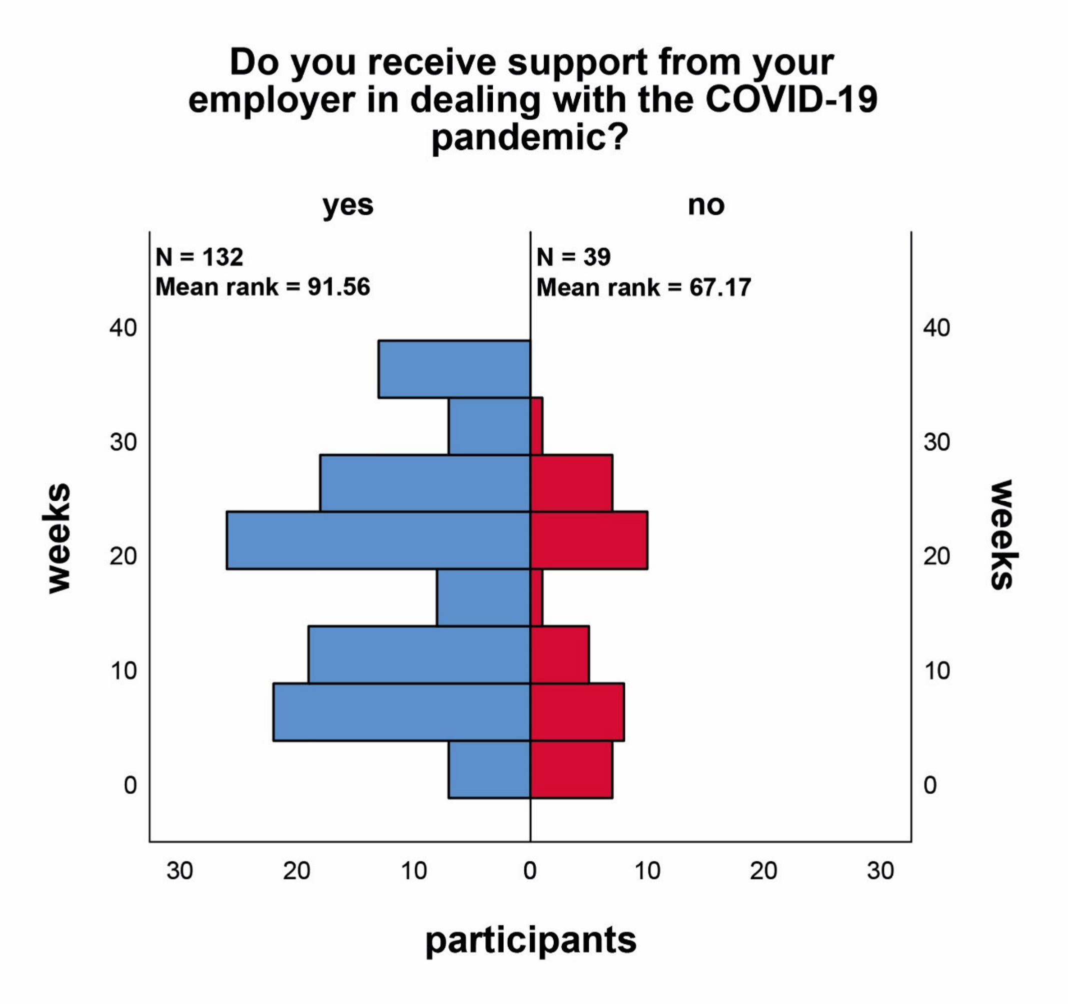

Supplement: Supplementary file 1 [file ijerph-18-12119-s001.zip › CareForMe_WHP_SupplementaryFigures_Legends_Rev1-edited.pdf]
