# Supplementary material for: Workplace Health Promotion and COVID-19 Support Measures in Outpatient Care Services in Germany: A Quantitative Study
Source: Int J Environ Res Public Health. 2021 Nov 18;18(22):12119. doi: 10.3390/ijerph182212119 (PMC8620567; doi:10.3390/ijerph182212119)
Supplement: Supplementary file 1 [file ijerph-18-12119-s001.zip › CareForMe_WHP_TableS1_Rev1-edited.pdf]

Supplement Table S1. Variables of the study instrument used to survey participants characteristics.

Item	Response options
To which gender identity do you identify?	Male/female/diverse
How old are you (in years)?	18–29/30–39/40–49/50–59/≥60
In which country was your father/mother born?	Germany/in another country
Please indicate the highest general school-leaving qualification, which you have achieved!	General secondary school/Intermediate secondary school/Specialised grammar school/Grammar school
Are you the supervisor for other employees?	Yes/No
Do you work in shifts?	Yes/No
