# Supplementary material for: Workplace Health Promotion and COVID-19 Support Measures in Outpatient Care Services in Germany: A Quantitative Study
Source: Int J Environ Res Public Health. 2021 Nov 18;18(22):12119. doi: 10.3390/ijerph182212119 (PMC8620567; doi:10.3390/ijerph182212119)
Supplement: Supplementary file 1 [file ijerph-18-12119-s001.zip › CareForMe_WHP_TableS2_Rev1-edited.pdf]

**Supplement Table S2.** Self-administered variables of the study instrument used in the analyses.

| Topics | Self-developed variables and response options | Items (n) |
|---|---|---|
| **Workplace health promotion** | • *Which of the following health promotion services are offered to you by your employer? (Nutritional counselling, cooking courses, breakfast with colleagues, free fruits, cost reductions and vouchers for fitness studios, sports classes e.g. fitness or yoga at workplace, back school courses, provision of company bicycles, courses on the topic of maintaining health, stress management courses, courses on dealing with violence/self-defence, addiction prevention (e.g., cessation seminars for smokers)* - is NOT OFFERED by employer and I would NOT USE it/is NOT OFFERED by employer but I would WANT it/is OFFERED but I DO NOT USE it/I USE this offer and it ALMOST DOES NOT HELP me/I USE this offer and it ALMOST DOES HELP me I am NOT SURE if this offer exists or not<br>• *What other offers would you like to see in order to better cope with the demands of your work?* – [free text] | 13 |
| **Use of health apps** | • *Have you ever used health apps for one or more of the following areas? (For example: nutrition, sport/pedometers, sleep, relaxation/stress reduction)* Yes/No/Don't know<br>• *If yes, how regularly have you used health behaviour app for nutrition/sport and pedometers/sleep and relaxation/stress reduction in the last 3 months?* never/rarely/sometimes/often/very often | 5 |
| **Break behaviour** | • *Has your break behaviour changed since the beginning of the COVID-19 pandemic?* - Yes/No/Don't know<br>• *How has your break behaviour changed? – [free text]*<br>• *Think about the last 4 weeks. How long were your actual breaks on average? – [free text]*<br>• *Do you have a break room available at work?* Yes/No/Don't know<br>• *Remember the time before the pandemic. Where did you (usually) spend your break? (Multiple answers possible) - car /break room/outdoors (e.g., park)/café, bakery/supermarket /at home/other place* | 5 |
| **COVID-19 support measures** | • *Do you receive support from your employer in dealing with the COVID-19 pandemic?* Yes/No<br>• *What support services for dealing with COVID-19 do you receive?* – [free text] | 2 |
